# Defining Quality of Life in the Children of Parents with Severe Mental Illness: A Preliminary Stakeholder-Led Model

**DOI:** 10.1371/journal.pone.0073739

**Published:** 2013-09-10

**Authors:** Penny Bee, Kathryn Berzins, Rachel Calam, Steven Pryjmachuk, Kathryn M. Abel

**Affiliations:** 1 Institute of Brain, Behaviour & Mental Health, University of Manchester, Manchester, United Kingdom; 2 Division of Clinical Psychology, University of Manchester, Manchester, United Kingdom; 3 Centre for Women’s Mental Health, University of Manchester, Manchester, United Kingdom; University of Granada, Spain

## Abstract

Severe parental mental illness poses a challenge to quality of life (QoL) in a substantial number of children and adolescents, and improving the lives of these children is of urgent political and public health concern. This study used a bottom-up qualitative approach to develop a new stakeholder-led model of quality of life relevant to this population. Qualitative data were collected from 19 individuals participating in focus groups or individual interviews. Participants comprised 8 clinical academics, health and social care professionals or voluntary agency representatives; 5 parents and 6 young people (aged 13–18 yrs) with lived experience of severe parental mental illness. Data underwent inductive thematic analysis for the purposes of informing a population-specific quality of life model. Fifty nine individual themes were identified and grouped into 11 key ‘meta-themes’. Mapping each meta-theme against existing child-centred quality of life concepts revealed a multi-dimensional model that endorsed, to a greater or lesser degree, the core domains of generic quality of life models. Three new population-specific priorities were also observed: i) the alleviation of parental mental health symptoms, ii) improved problem-based coping skills and iii) increased mental health literacy. The identification of these priorities raises questions regarding the validity of generic quality of life measures to monitor the effectiveness of services for families and children affected by severe mental illness. New, age-appropriate instruments that better reflect the life priorities and unique challenges faced by the children of parents with severe mental illness may need to be developed. Challenges then remain in augmenting and adapting service design and delivery mechanisms better to meet these needs. Future child and adult mental health services need to work seamlessly alongside statutory education and social care services and a growing number of relevant third sector providers to address fully the quality of life priorities of these vulnerable families.

## Introduction

Improving the lives of children born to a parent with severe mental illness (SMI) is of increasingly urgent political and public health concern [1]. Empirical work suggests that at least one quarter of adults admitted to UK adult acute inpatient settings are likely to have dependent children and that between 50–66% of people with severe mental illness may be living with children under the age of 18 [2], [3].

Although operational definitions of SMI vary [4], [5], the term has been defined from the user perspective to include schizophrenia, psychosis, bipolar disorder, severe mood disorders and personality and borderline personality disorders [6]. The burden placed on children living with parents with severe mental illness is substantial [2]. Children of parents with SMI are at greater risk of psychological and physical ill-health [7], [8], increased behavioural and developmental difficulties [7]–[9], educational underachievement [3], [10] and lower competency than their peers [11]–[14]. Compared to the children of healthy parents, children living with severe parental mental illness may also be exposed to greater material deprivation, increased caring responsibilities and a higher risk of child maltreatment and neglect [2], [15].

With increasing emphasis being placed on evidence-based healthcare, there is a pressing need to demonstrate the effectiveness and cost-effectiveness of interventions for these populations. The key challenge for services is in knowing when and how best to intervene. Of particular concern to health service commissioners may be the overwhelming lack of contemporary data pertaining to the clinical-and cost-effectiveness of community-based interventions aimed at improving children’s subjective quality of life. A systematic review of the literature finds current best evidence confined to poorer quality research prioritising researcher-led developmental or behavioural outcomes. Child-centred quality of life outcomes remain strikingly sparse [16].

The recognition that health and wellbeing refers to more than the mere absence of disease, has helped to elevate quality of life (QoL), and more specifically health-related quality of life (HRQoL) as an important clinical outcome for both adult and child services [17]. The term “quality of life” generally refers to an individual’s perception of their own life experience within the context of their personal goals, expectations, beliefs and perceptions [18]. Distinction is drawn between one dimensional measures that quantify satisfaction with a single life aspect and more multi-dimensional models that integrate satisfaction across a broader range of domains. One dimensional models often fail to reflect the full scope and complexity of quality of life judgments and can lack sensitivity to change. For this reason multidimensional models are generally preferred [19].

The nature and number of life domains assessed by multidimensional QoL models are not fixed phenomena. Nonetheless, most generic models are consistent in delineating five core life domains. These are: i) physical health, ii) emotional health, iii) material wellbeing, iv) environmental wellbeing and v) social function. Models that adopt a psychological or needs-based approach may also emphasise a unique contribution from self-actualisation and achievement [20].

Health-related quality of life prioritises domains that fall under the influence of healthcare systems, policy makers and providers [21]. Greater emphasis is often placed on HRQoL within health research and health economic evaluations, where the need to make resource allocation decisions between competing interventions for a disease, or between different categories of disease, has led to a policy preference for a common unit of outcome [22], [23]. Yet compared to adult constructs, child-centred quality of life models remain in a relatively early stage of development.

Within the UK, national policy initiatives with a broad perspective on children’s quality of life include the recently archived Every Child Matters agenda in England and Wales [24]; the Children’s and Young People’s Strategy in Northern Ireland [25] and the ‘Right of Every Child’ in Scotland [26]. Although derived from stakeholder consultation, these initiatives remain biased towards societal perspectives and to clinical and service outcomes more readily quantified through objective means. Subjective scales arguably offer a more direct approach to assessing children’s HRQoL, although early assessments developed purely from a biomedical perspective remain largely disease-specific [27]. More recently, generic child-centred measures have emerged, for example the GCQ [28], with potentially greater generalisability to non-clinical populations [19], [29].

A recent review has suggested that standard definitions of quality of life do not fully capture the experiences of children of parents with severe mental illness [30]. Children living with parents with severe mental illness encounter specific stressors related to disrupted life routines, repeated episodes of illness and hospitalisation of their parents, causing fracturing of family, academic and social lives. They also have poor mental health literacy and limited strategies for coping [3]. We sought to develop a new, stakeholder-led model of quality of life for children of parents with severe mental illness by using a ‘bottom-up’ qualitative approach.

## Methods

### Ethics

Favourable ethical review was obtained from the host institution's Research Ethics Committee (University of Manchester University Research Ethics Committee Ref: 10309) and the research panel of a national voluntary user organisation (ReTHINK Research Review Panel Ref: Bee). All participants provided signed, written consent. Signed, written parental consent was also obtained for any study participant aged less than 18 years.

Study participants were recruited by advertisement or email contact from outside of statutory health services. Service contact was not affected by study participation. Study information sheets were sent to all individuals who expressed an interest in the study at least 48 hours prior to written consent being taken. Members of the research team were available via email or telephone to answer any questions. Study participation was entirely voluntary and participants were free to withdraw at any time. Potential participants who declined to participate were not disadvantaged in any way.

### Participants and Recruitment

A qualitative approach to data collection was employed. In total, 19 individuals participated in the study, none of whom were previously known to the research team. Eligible participants were drawn from three separate stakeholder groups. Ethical requirements aimed at protecting participant anonymity demanded that each of these three participant groups were recruited from a different geographical area or via a distinct recruitment pathway. Table 1 presents aggregated, anonymised demographic data for the study sample.

**Table 1:** Demographic data for stakeholder sample (n = 19).

Characteristic	n (%) or median (range)
**Child participants (n = 6)**	
Male	2 (33)
Age in years	15 (13–18)
Mother with SMI	4 (66)
Parental Diagnosis:	
Bipolar Disorder	2 (33)
Major Depressive Disorder	2 (33)
Schizophrenia	1 (17)
Borderline Personality Disorder	1 (17)
**Parent participants (n = 5)**	
Male	1 (20)
Age of children	12 (10–17)
Parental Diagnosis:	
Bipolar Disorder	2 (40)
Major Depressive Disorder	2 (40)
Personality Disorder	1 (20)
**Professional participants (n = 8)**	
Male	3 (38)
Professional Background	
Mental Health Nursing	2 (25)
Clinical Psychology	1 (13)
Child Psychiatry	1 (13)
3^rd^ Sector user-led/voluntary organisations	4 (50)

It was recognised from the outset that the meaningful engagement of study participants would rely on an explicit acknowledgment of the ‘power’ and ‘stake’ present in each of the different stakeholder groups. The first group thus comprised eight higher power/lower stake individuals, in this case clinical academics with backgrounds in mental health nursing, child psychiatry and clinical psychology and professional representatives recruited via snowball sampling from clinical health and social care services, or through direct email correspondence with national user-led organisations, child-orientated charities and children’s mental health initiatives (e.g. Barnados, Young Minds, National Children’s Bureau, NSPCC).

The second and third groups comprised participants with potentially lower influence, yet higher stakes; in this case, parents and the children of parents with severe mental illness. A convenience sample of 5 parent participants (four mothers and one father) were independently recruited via advertisements placed on the website, email bulletins and Twitter feeds of a large national mental health user and carer organisation (ReTHINK Mental Illness). Each parent had at least one child under the age of 18 and experienced a severe, enduring mental illness: personality disorder (n = 1), bipolar disorder (n = 2) and major depressive disorder (n = 2). A further six young people with current lived experience of parental mental illness were recruited via convenience sampling from a young carers’ service in the South West of England. Primary parental mental health diagnoses, as reported by the families comprised bipolar disorder (n = 2), major depressive disorder (n = 2), schizophrenia (n = 1) and borderline personality disorder (n = 1). All child participants were approached indirectly by service managers and ranged in age from 13–18 years. Ethical and pragmatic constraints meant the views of younger children could not be collected. No study withdrawals occurred.

### Procedures

Data collection was undertaken by two researchers trained in qualitative methods (PB, KB). A third researcher accompanied PB for the duration of the professional focus group for the purposes of taking field notes and practical participant support. All data were collected between March-June 2011. Data collection ranged in duration from 35–90 minutes depending upon the nature of the stakeholder representation (Children 35–65 mins; Parents 40–80 mins; Professionals: 90 mins).

Individuals participated in either a focus group (professionals) or individual interview (children & parents), according to availability and personal preference. With the exception of 2 parents who completed telephone interviews, all data collection was conducted face-to-face in a workplace, service or community setting.

Data collection was driven by an open ended interview schedule that focussed discussion around participants’ general perceptions and understanding of quality of life, children’s and adolescents’ quality of life priorities, and the perceived relevance of these priorities to the children of parents with severe mental illness. Professional stakeholders were also asked to comment on their awareness of current quality of life models and the perceived validity of these models for children living with severe parental mental illness.

Interviews with adolescent stakeholders were informed by the principles of good practice for conducting social research with children [31]. To facilitate discussion between the interviewer and adolescent participants, an imaginary family-based scenario was used. This scenario described a child of non-specified age living with a parent with severe mental illness. Participants were asked to identify the key challenges and opportunities that they believed would be faced by this child and a series of blank cards and pens were made available to help the adolescents to formulate, organise and share their ideas. Completed cards were used as a prompt for further in-depth discussion and as a bridge back to the topics prioritised by the original interview schedule.

### Analyses

**Figure 1:**
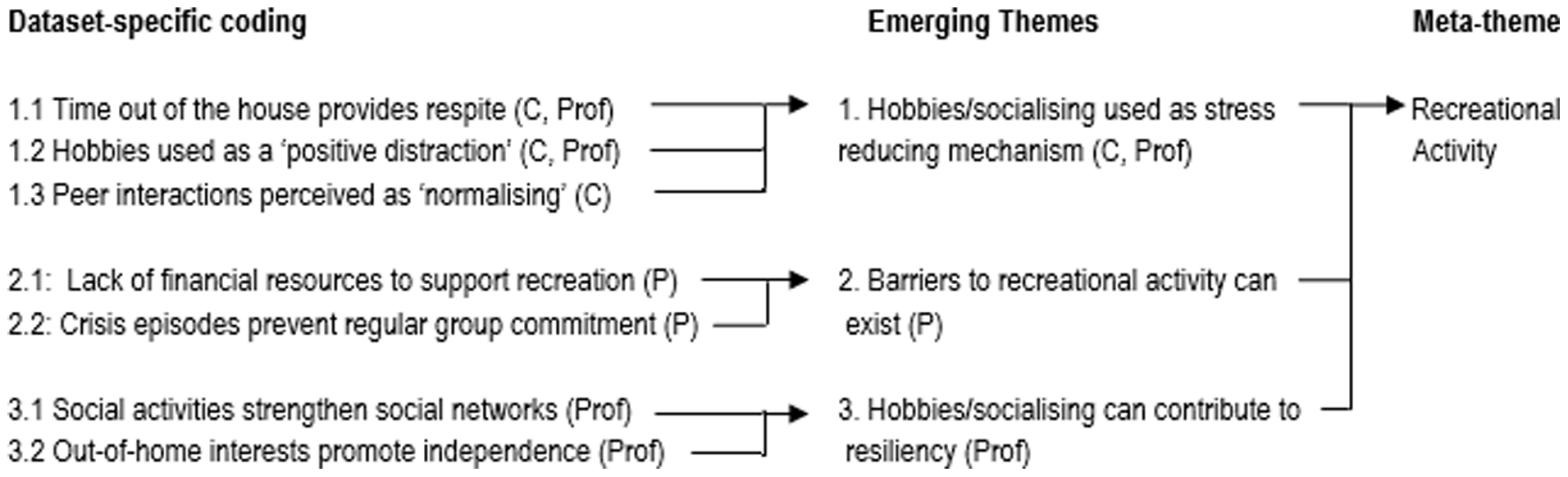
Example excerpt from the thematic analysis coding tree (C: Child, P: Parent, Prof: Professional).

The focus group and interviews were audio recorded and transcribed verbatim. Data underwent an inductive thematic analysis [32] for the purposes of informing a population-specific QoL model. Data were managed in MS-Word 2007 and analysed manually by PB. Each transcript was initially coded line by line according to the specific life experiences, goals or quality of life priorities that they were judged to represent. Codes that were judged to represent similar life facets, or different aspects of the same life facet, were then grouped together to identify emergent themes. Separate analyses were undertaken for each of the three stakeholder groups. The themes emerging from each dataset were then combined in a single, integrated coding tree and the themes that were judged to represent similar quality of life concepts or domains grouped together into a smaller number of ‘meta-themes’ (Figure 1). Each overarching meta-theme was mapped against a generic, UK-based children’s quality of life model (the Every Child Matters agenda [24]) in order to identify key similarities, and differences in scope. Independent verification of the emerging themes and meta-themes was provided by KB, who double coded the focus group transcript and a 25% sub-sample (n = 3) of the individual interviews. Coding discrepancies and differences in insights were discussed among the study team and new codes generated and incorporated into the analysis where appropriate. Although individual transcripts were not returned to participants for validity checking, all participants had the opportunity to comment on, and approve, the final coding tree. Ethical permissions that cautioned against data sharing between parents and children prohibited the deposition of data in publicly available resources.

## Results

Substantial overlap in quality of life concepts were observed across the three participant groups. Fifty nine individual themes emerged from the data, grouped into 11 key meta-themes (Table 2).

**Table 2:** Emergent themes grouped by overarching meta-themes.

Meta-theme	Child participants (n = 6)	Parent participants (n = 5)	Professional participants (n = 8)
**Emotional health**	Experience high anxiety regarding parents’ health; encounter daily stressors related to family conflict; engage with primary care services & use anti-depressant medication	Report common mental health problemsin children; express concern that SMIwill be inherited and want their childrento develop emotional resiliency	Consider children to be anxious about parents” health; report that children may fear developing their own mental health problem
**Social functioning**	Feel isolated from their peers	Perceive SMI to lead to behaviouralproblems in children	Acknowledge social withdrawal/distancing & potential behavioural problems in children
**Social relationship quality & support**	Value friendships for ‘normal’ interaction	Worry that children do not bring friends home; consider friendships important for ‘normal’ development	Believe children to be protected by accessible social support; perceive the presence of a supportive adult as key to emotional resiliency
**Recreational engagement**	Use hobbies/socialising as a stressreducing mechanism	Acknowledge barriers to recreationalactivity attendance; perceive recreationalactivity as beneficial tochildren’s development	Perceive recreational activities & social interaction to contribute to to children's resiliency
**Self-esteem**	Express a need for independence/autonomy	Believe children need strength ofcharacter to cope	Support services that promote confidence, aspiration & inner strength
**Problem-based** **coping**	Want practical solutions and caringsupport	Encourage children to develop effective practical skills in order to maintain their emotional health	Need services that foster empowerment, resilience & advocacy for children
**Mental health** **literacy**	Report confusion regarding a parent’serratic behaviour; perceive diagnostic/service information as important,report a lack of mentalhealth education	Highlight a need to explain mentalillness in an age appropriate manner.	Acknowledge the need for improved education about SMI & mental health services
**Family functioning**	Express concern for the stability of thefamily unit; derive enjoyment fromquality family time	Experience conflict between themselves, their children and other family members	Support service models that address whole family as well as individual needs
**Parental mental** **health symptoms**	Consider unpredictable parental responses difficult to manage; express widespread concern for parent’s safety & future	Undergo hospitalisation/unwanted separation from a child	Acknowledge that children’s basic needs are not always met and that children may assume caring responsibilities in times of crisis
**Parent-child interaction**	Strongly desire parental warmth & responsiveness; often feel like a target for parents’ hostile behaviour	Acknowledge erratic parenting,inconsistent boundaries and anger;perceive a lack of qualityinteraction & time together	Suggest that inconsistent parenting may negatively impact on a child; acknowledge that parents may be emotionally unavailable
**Economic resources**	Wish to alleviate everyday financialpressures; experience lack of food/hungeras a result of financial hardship;believe that low family incomedifferentiates them from their peers	Acknowledge lack of material possessions due to reduced income; report erratic provision of household resources and recreational provision	Perceive financial stability to be important for the whole family unit

**Figure 2:**
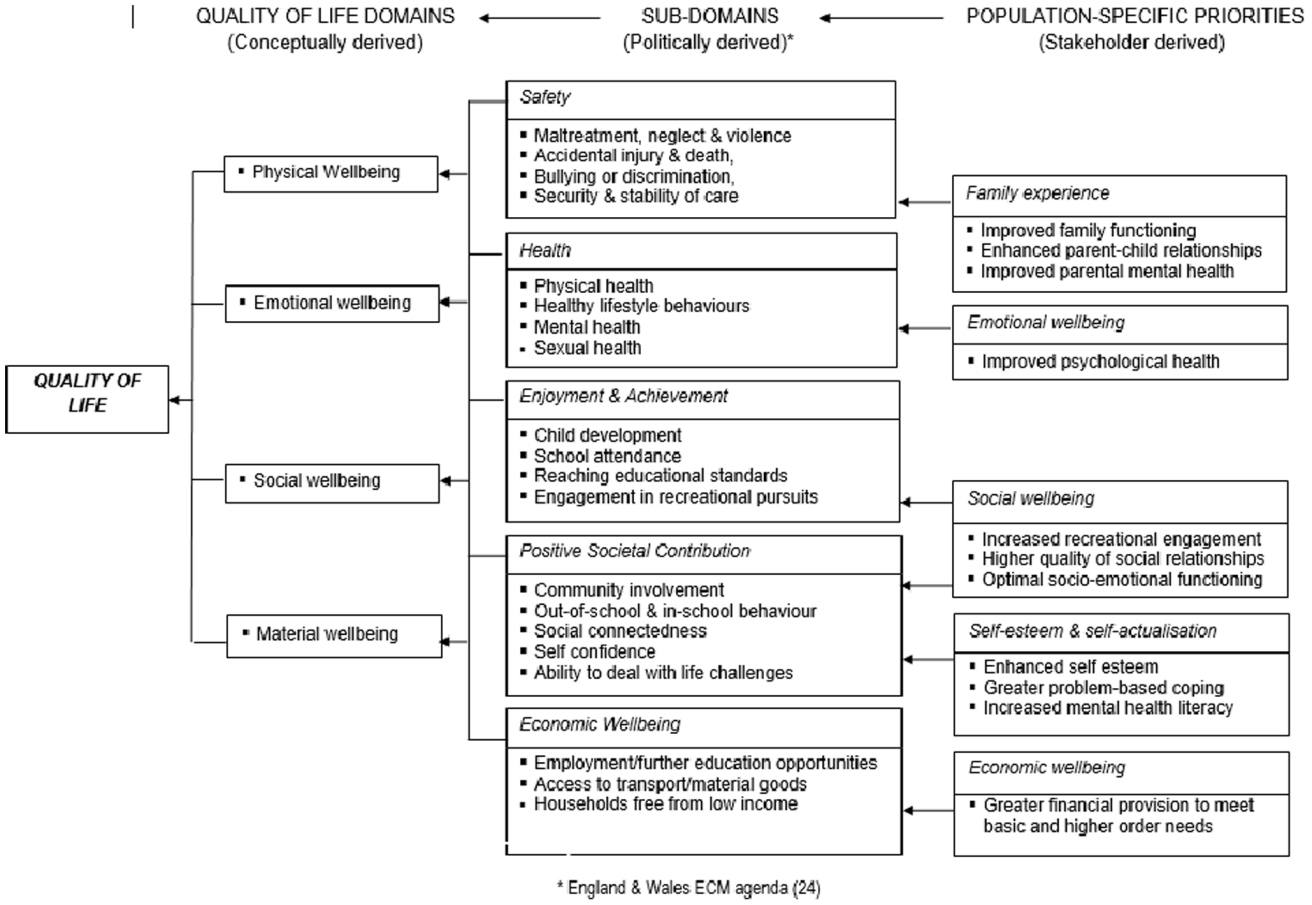
Conceptual QoL map for children of parents with mental illness.

Mapping each meta-theme against an existing UK child-centred quality of life framework revealed a multi-dimensional population-specific model that endorsed, to a greater or lesser degree, the core life domains prioritised by generic models (Figure 2). Quality of life in the children of parents with severe mental illness was judged to encompass and be underpinned by five key quality of life domains:

- Children’s emotional wellbeing
- Children’s social wellbeing
- Children’s economic wellbeing
- Children’s family contexts and experiences
- Children’s self-esteem and self-actualisation

These five QoL domains and the meta-themes that mapped onto them are discussed in further detail below.

### Children’s Emotional Wellbeing

Children’s emotional wellbeing was endorsed by one meta-theme advocated by all three participant groups. Both professional and child discourse focused heavily on children’s propensity to feel anxious or depressed about their parent’s mental health condition, and consequently to develop clinically significant mental health symptoms of their own:

*“I don’t know really it just … kind of affected me slightly mentally, having to deal with that, having to deal with what she’s like. Like, past like attempts of her trying to take too many pills, like, and sort of how to keep her calm. It’s hard. The doctor’s said I’m depressed.”*

(Child stakeholder, 15 yrs, Mother with bipolar disorder)

Parents identified multiple instances in which they perceived their children’s emotional wellbeing to be negatively influenced by the family’s experience of living with severe mental illness. They confirmed periods in which their children had been treated for, or displayed symptoms of, anxiety and depression and expressed high concern that their own mental illness had contributed to these difficulties. Parents often feared an increase in the future severity of their children’s mental illness with genetic transmission, behavioural mimicking and increased psycho-social stress all being postulated as possible causes:

*“My son says, ‘I can accept it mum’, but, but, what also happens is, they adopt a different persona, it rubs off on him to a certain degree, and he becomes irritated as well.”*

(Parent stakeholder, Mother with depression)

### Children’s Social Wellbeing

Children’s social wellbeing was endorsed by three different yet inter-related meta-themes relating to i) children’s socio-emotional functioning and behaviour, ii) children’s social relationship quality and iii) children’s recreational activity engagement.

The first meta-theme, children’s socio-emotional functioning and behaviour, encompassed children’s own perceptions of social isolation alongside parent’s and professional’s perceptions of children’s internalising and externalising symptoms. Children described how they often felt emotionally and functionally separated from their peers, primarily due to the dissimilarities that they experienced in their home environments, levels of family responsibility and daily routines. Professionals and parents confirmed this perspective. Professional stakeholders described observing tangible differences in children’s opportunities and propensity for social interaction, whilst parents’ focussed on the difficulties that they had perceived in fostering and nurturing town child’s independence. Both parents and professionals expressed concern that severe parental mental illness could contribute to social withdrawal and behavioural dysfunction in younger generations:

*“If I'm punching the wall, say, or I scream or just get so angry, she curls over ….she’s just not there, not there emotionally I mean”*

(Parent stakeholder, Mother with bipolar disorder)

The second meta-theme (children’s social relationship quality) encompassed multiple references and themes relating to the acquisition and maintenance of positive peer relationships. Both parent and child stakeholders emphasised the value that they placed on friendship, explicitly acknowledging its role as a conduit to normative, social interaction. Parents in particular identified peer relationships as a core component in, and proxy indicator of, healthy childhood development. Professional perspectives over-lapped with the parental stance but uniquely also underscored the importance of friendship as a necessary source of social support. High quality social support delivered within the context of an established and trusting social relationship was identified as key to all children’s quality of life, and particularly important in enhancing children’s resilience to parental mental illness:

*“Children need some-one who they can talk to, share with. If they have just one person who they are close to, who they trust, then they will have that support. This is all part of resilience, one of the ways they might get help, feel better.”*

(Professional stakeholder, National Children’s Charity)

The third meta-theme, children’s recreational activity engagement was advocated by all three stakeholder groups. Children’s engagement in out-of-home activities was perceived to both offer respite from family stressors and provide regular and ongoing opportunities for social, cognitive and physical development. Parents were explicit in recognising that young caring responsibilities and family financial hardship often prevented children from engaging in social or leisure pursuits, and that more meaningful involvement in such activities would potentially enhance their children’s quality of life. Professionals focussed on the potential role of recreational activity in enhancing children’s resiliency to severe parental mental illness whilst children similarly conceived recreational activities as a useful stress-relieving mechanism and a potentially effective means by which to integrate with peers:

*“Social, creative, miscellaneous type things, so my good day would be doing anything like that, playing the piano, going out with my friends, helping other people out, that would be part of my day.”*

(Child stakeholder, 17 yrs, Mother with psychosis)

### Children’s Economic Wellbeing

Economic wellbeing was endorsed by one meta-theme that encompassed a range of material, emotional and social needs. All three participant groups upheld financial stability and economic resources as a central factor in determining children’s quality of life, with multiple benefits emanating from a families’ capacity to meet children’s short and long term requirements. Financial security was deemed vital for the purposes of meeting children’s basic needs, such as food provision, as well as their higher order needs such regular engagement in family recreation. Economic instability was additionally identified by children as a key source of stigma and a unique barrier to social integration with their peers:

*“And mum she just says that, “I don’t have any money at all,” and so we literally have no food in our house, so I don’t really eat. My mum doesn’t have a fridge freezer, so we don’t have the normal things, things that everyone else would have…its not something I normally share.”*

(Child stakeholder, 14 yrs, Mother with psychosis)

### Children’s Family Contexts and Experiences

Children’s family contexts were endorsed by three meta-themes relating to i) parental mental health symptoms, ii) family functioning and iii) the quality of parent-child relationships.

Alleviating parental mental health symptoms was the main priority of all of the children we consulted. Across all three stakeholder groups, participants described a level of unpredictability in parents’ behaviour that had substantial impact on children’s sense of security and emotional wellbeing. Parents’ perspectives provided support for this observation by focussing predominantly on the potential disruption caused by crisis episodes and the absence of parental contact during periods of hospitalisation. Yet, for other stakeholders, the potential effects of severe parental mental illness extended beyond this notion to encompass more routine aspects of domestic function. Both professional and child stakeholders described prolonged episodes of parental dysfunction during which parenting could become difficult and children’s needs may be less likely to be met:

*“She may not be able to depend on her mum as much as she used to and she’ll have to, kind of, grow up a bit more. When her mum’s ill, a lot really, sometimes she may have to put her mum in front of her, of what she wants and needs”*

(Professional stakeholder, Adult Mental Health Services)

Adequate family functioning was identified as the second meta-theme in the family context and experience domain and was consistently emphasised as a key contributor to children’s quality of life judgements. Discussing the potential for family conflict, professional stakeholders focussed on the broader relevance of family experience to service development and the need to deliver family interventions capable of enhancing family communication and cohesion. Drawing directly on personal experiences, parents and children reflected a similar stance, albeit from a differnet perspective. Both parents and children described tension and breakdowns in intra and inter-family relationships, often with a negative outcome for children’s sense of security and family belonging:

*“We don’t have much contact with them anymore. My auntie used to come but she doesn’t now. I don’t see her now. If my mum’s not well, well, it’s …difficult. Dad tried when he was there but he’d get you know, upset and angry… sometimes he’d go to his room.”*

(Child stakeholder, 17 yrs, Mother with psychosis)

As part of the third meta-theme, the quality of the interaction between children and their parents, children specifically described the enjoyment they derived from spending ‘ordinary’ time within their families. Some children described times during which their parent was inexplicably angry or hostile towards them, and contrasted this with their desire to engage more frequently in warm and positive interactions with their parents. Parent and professional participants also highlighted the potential for parental behaviour and parenting routines to become erratic, recognising that this was often to the detriment of quality time spent with children.

*“My mum being happy, yes, seeing my mum have a smile on her face. Doing things together, even if it is going out, like walking down to the chip shop to go and get some chips, that would make me happy …”*

(Child stakeholder, 13 yrs, Mother with personality disorder)

### Children’s Esteem and Self-Actualisation

The final domain, children’s esteem and self-actualisation, was endorsed by three meta-themes relating to i) children’s self-esteem, ii) children’s problem-based coping and iii) children’s levels of mental health literacy.

Children’s self-esteem was considered by all three stakeholders to make a key contribution to children’s subjective appraisals of quality of life. Self-confidence, strength of character and personal achievement were viewed as inter-linked concepts underpinning self esteem in all children, but uniquely so in the children of parents living with severe mental illness. Parents focused primarily on the need for their children to develop and display a ‘strength of character’ in order to cope with the practical and emotional challenges presented by their illness and the impact of stigma on their day to day lives. Professionals similarly emphasised the value of fostering children’s self-confidence, specifically emphasising its role in promoting childhood resiliency, nurturing children’s short and long term aspirations and increasing the probability of children achieving positive life outcomes:

*It’s about accepting…not accepting it in a sort of negative way but appreciating just how well they’re doing to be coping with it, building up their own confidence about how much they can do.”*

(Professional stakeholder, 3^rd^ Sector Representative)

Children’s discourse reflected both parental and professional perspectives. Self-esteem was frequently identified by children as both a positive contributor to their own quality of life judgements, and as a personal attribute negatively influenced by their family circumstance. Perceived conflict was evident between children’s desires’ to maintain family and caring responsibilities, and their need to engage in out of home activities designed to re-affirm personal skills and self-worth. Children remained consistent in identifying personal autonomy as an underlying component of their self-worth and an important contributor to future life quality enhancement:

*“To feel better about myself I guess, to go to college next year maybe, to know I could do that, have a good job. My sister went, she went ‘cos I was there. I don’t have that, I need mum to get better first…. to be able to go, to go and know she’s OK.”*

(Child stakeholder, 17 yrs, Mother with psychosis)

The second and third meta-themes in this domain (children’s problem-based coping skills and mental health literacy) delineated two specific mechanisms through which resiliency to severe parental mental illness could most effectively be enhanced. All three stakeholder groups identified children’s individual capacities for problem-based coping as an important determinant of their emotional wellbeing and by implication, a key component in their subjective quality of life appraisals. Specific strategies thought to impart practical solutions to the challenges faced by this population included the instigation and maintenance of formal and informal support networks and the enhanced availability of advocacy services:

*“Just to know what to do…where to get help when its needed, Actual help, useful help, to help us cope day to day, to let me go out or to show me to manage mum’s money, to support me ….problems that might sometimes seem small,. or perhaps smaller if they were solved.”*

(Child stakeholder, 14 yrs, Mother with psychosis)

Low mental health literacy was uniquely and consistently identified as exerting a negative impact on children’s abilities to cope with and respond to their parent’s mental illness. Child participants reported multiple sources of confusion that included a lack of awareness regarding their parent’s mental health diagnosis, poor levels of understanding about their parent’s behaviours, and a lack of education regarding mental health service delivery and implementation. Parents and professionals also identified a pressing need for clear, child centred, age-appropriate information:

*“Children don’t always understand what is going on, they can get frightened or confused by people coming in and out of their homes. They need to know who these people are, and why they are there. We try to help with that, that’s part of what we do…. it can make a difference, a big difference to the way a child feels.”*

(Professional stakeholder, 3^rd^ Sector Representative)

## Discussion

The current study drew on broad stakeholder perspectives to inform a conceptual model of quality of life that better reflects the needs and life priorities of contemporary British children living with parents diagnosed with a severe mental illness. This QoL model is innovative because it is grounded in children’s lived experience rather than being service driven. A total of five key domains and 11 sub-domains (meta-themes) were identified, all of which could be mapped to one or more components of existing quality of life models.

Three priorities specific to this vulnerable group of children were observed: alleviation of parental mental health symptoms, improved problem-based coping skills and increased mental health literacy. These elements appear central to children’s psychological resilience, either enhancing their wellbeing or protecting them from the influence of risk factors by enabling them to interact better with their family and home environments. Similar priorities have been reported by other user consultation exercises [2], [33] and empirical work [34]. Studies specifically focussing on young carers report these children to have multiple responsibilities including looking after other members of the family, mediating family conflict and seeking out help for the ‘looked after’ person [35]. Such observations provide one explanation for why effective coping strategies and particularly those based on problem-focused approaches may have been endorsed by participants as a key mechanism through which children can be empowered to maintain long term emotional health.

Family and parental experiences remain an important component of children’s life experiences and a key contributor to their quality of life judgments, particularly when parents suffer from severe mental illness [30], [34]. Interventions that target parental mental health or family function, and monitor treatment effects in terms of parental mental health symptoms, parenting behaviours or child-centred psychopathological outcomes are thus likely to be relevant to children’s quality of life, particularly where children and parents with severe mental illness live together.

Notably, our stakeholder consultants failed to endorse three key quality of life influences currently upheld by national child-centred policy initiatives such as the England & Wales Every Child Matters Agenda [24]. These elements comprised children’s safety (defined in terms of child neglect, maltreatment or violence), children’s cognitive and educational development and children’s physical and sexual health. Ultimately, the omission of these influences from our data may reflect a bias towards healthy participants recruited from non-clinical settings. Alternatively, it may be that these factors do not sit well within children’s subjective models of quality of life. Such issues may also be minimised by parents and professionals because they comprise somewhat more complex and less easily manageable problems. Self-perceived health-related quality of life is distinct from an individual’s physical health status and caution should always be taken when interpreting these outcomes as proxy indicators of children’s quality of life.

Child-centred quality of life data have been collected in previous studies of vulnerable children, mainly in families where risk is derived from multiple (social) factors rather than primarily as a result of parental mental ill health. Approximately 2% of UK families are reported to suffer the combined effect of parental mental illness, low income, lower educational attainment and poor housing; and this group is one of the most vulnerable in society [36], [37]. Many multi-risk families are characterised and defined by social deprivation indices rather than by mental illness, therefore missing substantial numbers of children experiencing parental ill health. Although valuable lessons may be learnt from the child-centred QoL data collected from these samples, the specific needs of the two groups are likely to differ. Children living in economically deprived families will not necessarily be acting as informal carers and will not routinely experience chaotic and sometimes potentially frightening behaviours or repeated separation from their parents during crisis periods and parental hospitals admissions.

Current UK child-centred policy considers a broad spectrum of child-centred outcomes as suggested by the multidimensional approach taken by the Every Child Matters agenda [24]. This agenda highlights specific quality of life domains relevant to child health (e.g. physical & emotional health), safety (e.g. accidents, injury & maltreatment, stability of care), enjoyment and achievement (e.g. cognitive development, school and recreational engagement), making a positive societal contribution (e.g. social behaviour, self-esteem and coping) and economic wellbeing (e.g. access to material resources or income).

Appropriately enough, UK policy continues to advocate greater support for families and children affected by mental illness, including working directly with children. Families have different and multi-faceted needs, requiring a multi-agency approach in which child and adult mental health services work seamlessly together, alongside statutory education and social care services and a growing number of relevant third sector services. Outreach services linking the UK NHS to the community and community based services involving other agency organisations may also have a role. Recent evaluations suggests that UK models of joint agency working may be better developed for drug and alcohol services, where a national requirement to monitor the numbers of adult service users with children has served as an effective driver for family needs assessments and child-focussed social care [38].

### Strengths and Limitations

The current study sought to develop a new, stakeholder-led model of quality of life for children of parents with severe mental illness using a ‘bottom-up’ qualitative approach. We acknowledge that our participants represent a relatively small, convenience sample with views that may not be generalisable to all parent, child and service provider perspectives. Due to practical and ethical constraints, the current research recruited young people aged between 13–18 years from the same geographical area. Many children of parents with severe mental illness remain invisible to services and it is possible that data saturation has not yet been achieved. Child and parent participants consented voluntarily to interview and may thus have been more willing to discuss personal experiences compared to other individuals living in less desirable circumstance. Considerable effort was spent in ensuring that all interviews proceeded in an open and non-judgmental way, However it is still possible that specific experiences may not have been discussed and thus may not be fully represented in our preliminary stakeholder-led quality of life model. The inclusion of participants with relevant professional backgrounds facilitated the triangulation of multiple stakeholder perspectives. However, quality of life of life remains a subjective concept [18] and the extent to which adult participants can meaningful contribute to child-centred quality of life constructs remains a matter for debate.

### Future Work

Although our results are preliminary, they suggest that future population specific quality of life measures should take account of the broader values endorsed by our study participants. We recommend a comprehensive and systematic review of children’s needs and experiences, aimed at delineating the quality of life priorities of different groups. Full and due consideration should be given to determining the relevance of existing quality of life measures to the children of parents with severe mental illness, and to establishing potential differences in the quality of life priorities of children of different ages, not least because children’s quality of life judgments are likely to be directly influenced by the cultural and temporal contexts in which they occur.

Where necessary, a larger programme of work should be undertaken to develop and validate novel and age-appropriate HRQoL measures for these populations. The challenge then remains to establish how, and to what extent, these self-appraised quality of life outcomes may translate into future service design and intervention.
